# Weight and Metabolic Outcomes in Naïve HIV Patients Treated with Integrase Inhibitor-Based Antiretroviral Therapy: A Systematic Review and Meta-Analysis

**DOI:** 10.3390/jcm12113644

**Published:** 2023-05-24

**Authors:** German Valenzuela-Rodriguez, Carlos Diaz-Arocutipa, Jaime A. Collins, Adrian V. Hernandez

**Affiliations:** 1 Clinica Delgado, Servicio de Medicina Interna y Cardiologia, Lima 15074, Peru; 2 Unidad de Revisiones Sistematicas y Meta-Analisis (URSIGET), Vicerrectorado de Investigacion, Universidad San Ignacio de Loyola, Lima 15024, Peru; 3 Instituto de Evaluación de Tecnologías en Salud e Investigación—IETSI, EsSalud, Lima 15072, Peru; 4 Servicio de Infectologia, Hospital Nacional Guillermo Almenara Irigoyen-EsSalud, Lima 15033, Peru; 5 Health Outcomes, Policy, and Evidence Synthesis (HOPES) Group, University of Connecticut School of Pharmacy, Storrs, CT 06269, USA

**Keywords:** integrase inhibitors, weight, cholesterol, triglycerides, meta-analysis

## Abstract

Background: The use of integrase inhibitor-based antiretroviral therapy could be associated with worse weight and metabolic outcomes in patients with HIV infection. Methods: PubMed, EMBASE, and Scopus were searched from inception to March 2022. We selected randomized controlled trials (RCTs) comparing integrase inhibitors with other antiretroviral classes (efavirenz-based or protease inhibitor-based therapies) in naïve HIV patients. Random effects meta-analysis was used to assess the effects of integrase inhibitors vs. controls on weight and lipid outcomes. Effects were described as mean differences (MD) and their 95% confidence intervals (CI). Certain pieces of evidence (CoE) were evaluated using the GRADE methodology. Results: Six RCTs (n = 3521) were included, with patients followed up between 48 and 96 weeks. The use of integrase inhibitors in comparison with other antiretroviral classes was associated with an increase in weight (MD 2.15 kg, 95%CI 1.40 to 2.90, I^2 ^= 0%, moderate CoE), and decreases in total cholesterol (MD −13.44 mg/dL, 95%CI −23.49 to −3.39, I^2^ = 96%, low CoE), LDL cholesterol (MD −1.37 mg/dL, 95%CI −19.24 to −3.50, I^2 ^= 83%, low CoE), HDL cholesterol (MD −5.03 mg/dL, 95%CI −10.61 to 0.54, I^2 ^= 95%, low CoE), and triglycerides (MD −20.70 mg/dL, 95%CI −37.25 to −4.15, I^2 ^= 92%, low CoE). There was a high risk of bias in two RCTs and some concerns about bias in two RCTs. Conclusions: In HIV patients, the use of integrase inhibitor-based therapy in comparison with protease inhibitor- or NNRTI-based therapy was associated with a small increase in weight and small decreases in lipid serum levels.

## 1. Introduction

Weight gain is common among patients infected with the human immunodeficiency virus (HIV) who initiate antiretroviral therapy (ART), and it could be associated with minor mortality among individuals who are initially underweight or of normal weight [1]. Factors associated with obesity are shared between people with HIV (PWH) and the general population [2]. However, ART initiation reverses the catabolic state found in malnourished patients, reduces circulating inflammatory biomarkers, and can improve appetite and nutrient absorption [2,3].

There is evidence about the different effects of ART drugs on weight changes. For example, nucleoside reverses transcriptase inhibitors (NRTIs) and non-nucleoside reverse transcriptase inhibitors (NNRTIs) have not been associated with differential weight gain. However, some studies suggest that efavirenz and tenofovir disopropil may inhibit weight gain [4,5,6].

Integrase strand transfer inhibitors (INSTI; e.g., raltegravir, elvitegravir, dolutegravir, bictegravir, and cabotegravir—used in combination with rilpivirine—) are now recommended as first-line treatments for PWH, probably because of their good tolerability profile and genetic barrier to HIV drug resistance (the resistance barrier of bictegravir and dolutegravir is better than that of raltegravir and elvitegravir) [7]. Nevertheless, several single-center sites, cohort studies, and clinical trials reported more significant weight gain among persons receiving INSTI-based ART regimens for initial therapy as compared to protease inhibitors (PIs) and NNRTI-based regimens [8,9,10,11,12,13,14,15,16]. Moreover, the first published studies with INSTIs, mostly with raltegravir, found a neutral variation in lipids [17].

We systematically evaluated the weight and metabolic effects of INSTI-based ART in comparison to NNRTI- or PI-based ART in randomized controlled trials (RCTs) conducted in naïve HIV patients.

## 2. Materials and Methods

This systematic review was reported following the 2020 Preferred Reporting Items for Systematic Reviews and Meta-Analyses (PRISMA) guidelines [18]. The protocol and the systematic review were not previously registered.

### 2.1. Searches

Using the electronic databases Pubmed, EMBASE, and Scopus until March 2022, we identified all published RCTs that reported weight as an outcome in PWH treated with antiretrovirals of the INSTI class. We developed the search strategy without any language restriction (Table S1). We also manually searched reference lists of all retrieved articles.

### 2.2. Study Selection

Two reviewers (G.V. and J.C.) independently made study selections, with disagreements solved through discussion and by another reviewer (C.D.). Studies were considered potentially eligible if they met the following criteria: (I) phase III RCTs in naïve HIV patients; (II) reported quantitative values of weight or lipid outcomes; (III) compared the effects of integrase inhibitors with another type of therapy: protease inhibitors and NNRTI. All of the data was obtained from the original paper with pre-defined follow-up times, and we did not assess outcome data from extension RCTs (i.e., when patients were not randomized anymore).

### 2.3. Outcomes

We pre-specified weight, total cholesterol, LDL cholesterol, HDL cholesterol, and triglycerides as outcomes. We did not have a restriction on the time of follow-up.

### 2.4. Data Extraction

Two reviewers (G.V. and C.D.) independently extracted data from the included RCTs. The following information was obtained from individual studies: author, year of publication, sample size, baseline patient characteristics (age, sex, weight), time of follow-up, INSTI drug, dose and duration, comparator drug, dose and duration, and outcomes per arm: weight, total cholesterol, LDL cholesterol, HDL cholesterol, and triglycerides.

### 2.5. Risk of Bias Assessment

Two reviewers (G.V. and C.D.) independently assessed the risk of bias (RoB) using the Cochrane RoB 2.0 tool [19]. Five domains were evaluated: the randomization process (D1), deviations from intended interventions (D2), missing outcome data (D3), measurement of the outcome (D4), and selection of the reported result (D5). Each domain was rated as low, high, or having some concerns using a pre-defined algorithm, and then each RCT was rated as being high, low, or having some concerns about bias (high when there was at least one domain at high risk of bias; some concerns when at least one domain was rated at some concerns but no domain was rated at high risk of bias).

### 2.6. Statistical Analyses

All meta-analyses were conducted using a random-effects model and the inverse variance method. Effects of INSTI on outcomes were described as mean differences (MD) and their 95% confidence intervals (95% CIs). We used the Paule-Mandel method for the calculation of between-study variance (tau^2^) [20] and the Hartung-Knapp method for the adjustment of 95% CIs [21]. Statistical heterogeneity was evaluated using the I^2^ statistic and was defined as follows: I^2^ > 30% low, 30–60% moderate, and >60% high heterogeneity [22]. We evaluated small study effects with Egger’s test when there were data from 10 studies or more per outcome [23]. We used the *meta* package from R 4.1.2 (www.r-project.org, accessed on 1 March 2022) for all statistical analyses.

### 2.7. Certainty of Evidence

The certainty of evidence (CoE) was assessed using the GRADE methodology (www.gradeworkinggroup.org, accessed on 1 March 2022). The CoE per outcome was based on the evaluation of five characteristics: RoB, imprecision, inconsistency, indirectness, and publication bias. A description of CoE was shown in the summary of findings (SoF) tables using GRADEpro software version 2021 (McMaster University and Evidence Prime; www.gradepro.org/, accessed on 1 March 2022).

## 3. Results

### 3.1. Study Selection

**Figure 1:**
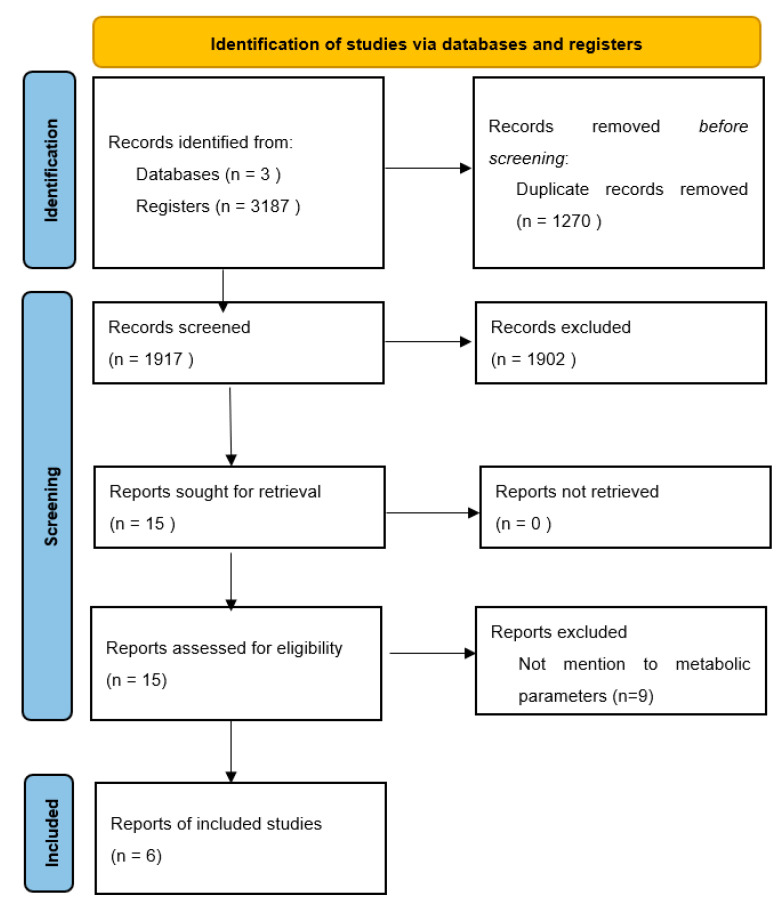
Flowchart of study selection.

A total of 3187 abstracts were evaluated. After excluding 3172 abstracts, we retrieved the full texts of 15 studies for detailed evaluation (Figure 1). Nine studies were excluded because of a lack of quantitative values in metabolic outcomes. At the end of the process, the information from 3521 patients extracted from six RCTs [24,25,26,27,28,29] was included in the final analysis.

### 3.2. Study Characteristics

Our six included RCTs were published between 2009 and 2020. Drugs being compared included raltegravir versus efavirenz [24], elvitegravir versus atazanavir-ritonavir [25], elvitegravir versus efavirenz [26], dolutegravir versus efavirenz [27,28], and dolutegravir versus darunavir [29]. (Table 1). The number of patients evaluated across RCTs ranged from 484 to 1053. Follow-up times among RCTs were between 48 and 96 weeks. Four studies were multicentric, and two were conducted in a single country (Cameroon and South Africa). Patients had a mean age of 30 years, and the proportion of males varied from 31.7% to 91.8% (Table 1).

### 3.3. Risk of Bias Assessment

**Figure 2:**
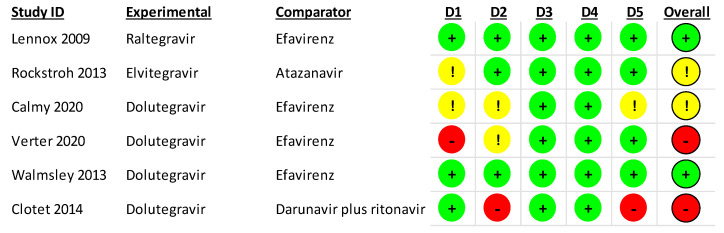
RoB2 assessment of included randomized controlled trials. RoB2 domains: D1: randomization process; D2: deviations from intended interventions; D3: missing outcome data; D4: measurement of outcome; D5: selection of the report result. Red (-) describes a high risk of bias; yellow (!) describes some concerns about bias; and green (+) describes a low risk of bias.

The risk of bias in RCTs is shown in Figure 2. In the domain of randomization, one study had high concerns about bias where treatment allocation was not masked to investigators or patients [27], and two had some concerns [25,26]. In the domain of deviations from the intended interventions, one study had high concerns of bias where no masking was done in the study [29], and two had some concerns [26,27]. In the domain of the selection of the reported results, one study had a high risk of bias where there were described deviations from the pre-specified analysis [29], and one had some concerns about bias [26].

### 3.4. Effects of INSTIs on Weight

**Figure 3:**
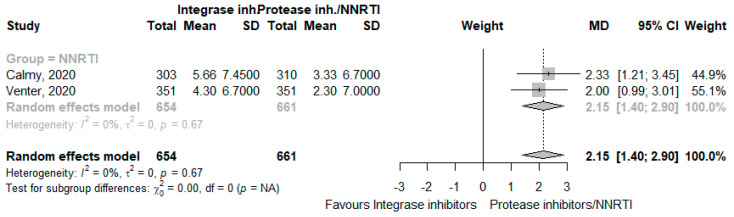
Effects of integrase inhibitors (Integrase inh.) on weight compared to control: protease inhibitors (Protease inh.) or NNRTI. Squares represent the mean difference (MD) of each individual RCT, horizontal lines represent the 95% confidence intervals (CI) of the MD, and diamonds are the MD of the overall random effects meta-analysis.

Two RCTs reported variations in weight gain [26,27]. The use of INSTI increased weight by 2.15 kg (95% CI 1.40 to 2.90, I^2^ = 0%, moderate CoE; Figure 3 and Table S2).

### 3.5. Effects of INSTIs on LIPIDS

**Figure 4:**
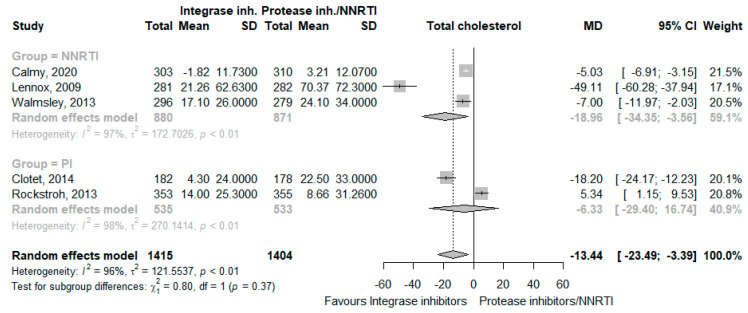
Effects of integrase inhibitors (Integrase inh.) on total cholesterol compared to control, stratified by type of control: protease inhibitors (Protease inh.) or NNRTI. Squares represent the mean difference (MD) of each individual RCT, horizontal lines represent the 95% confidence intervals (CI) of the MD, and diamonds are the MD of the random effects meta-analysis per stratum and overall.

**Figure 5:**
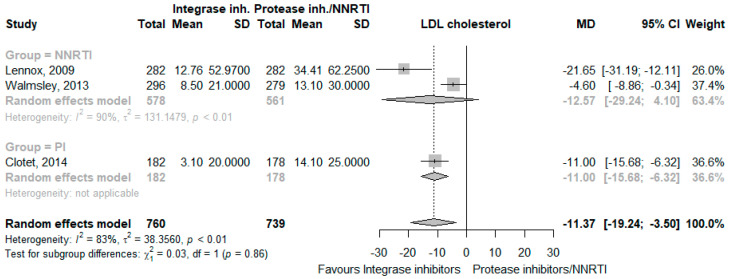
Effects of integrase inhibitors (Integrase inh.) on LDL cholesterol compared to control, stratified by type of control: protease inhibitors (Protease inh.) or NNRTI. Squares represent the mean difference (MD) of each individual RCT, horizontal lines represent the 95% confidence intervals (CI) of the MD, and diamonds are the MD of the random effects meta-analysis per stratum and overall.

**Figure 6:**
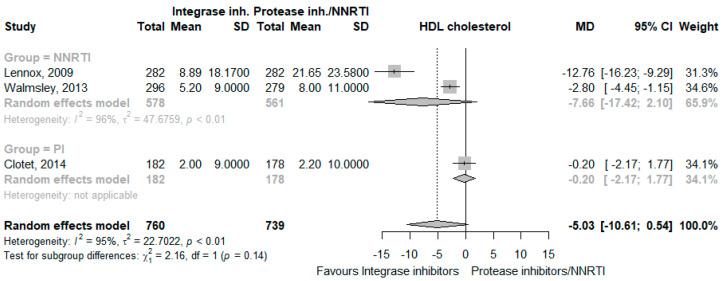
Effects of integrase inhibitors (Integrase inh.) on HDL cholesterol compared to control, stratified by type of control: protease inhibitors (Protease inh.) or NNRTI. Squares represent the mean difference (MD) of each individual RCT, horizontal lines represent the 95% confidence intervals (CI) of the MD, and diamonds are the MD of the random effects meta-analysis per stratum and overall.

**Figure 7:**
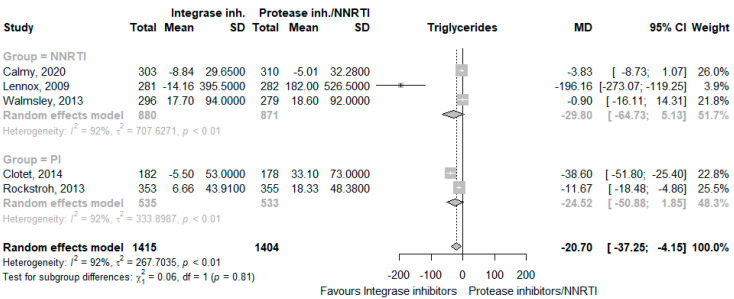
Effects of integrase inhibitors (Integrase inh.) on triglycerides compared to control, stratified by type of control: protease inhibitors (Protease inh.) or NNRTI. Squares represent the mean difference (MD) of each individual RCT, horizontal lines represent the 95% confidence intervals (CI) of the MD, and diamonds are the MD of the random effects meta-analysis per stratum and overall.

The use of INSTI decreased total cholesterol levels (MD −13.44 mg/dL, 95% CI −23.49 to −3.39, I^2^ = 96%, five studies [24,25,26,28,29], Figure 4), LDL cholesterol levels (MD −11.37 mg/dL, 95% CI −19.24 to −3.50, I^2^ = 83%, three studies [24,28,29], Figure 5), HDL cholesterol levels (MD −5.03 mg/dL, 95% CI −10.61 to 0.54, I^2^ = 95%, three studies [24,28,29], Figure 6), and triglycerides (MD −20.70 mg/dL, 95% CI −37.25 to −4.15, I^2^ = 92%, five studies [24,25,26,28,29], Figure 7). The CoE for all lipid outcomes was low (Table S2).

## 4. Discussion

### 4.1. Main Findings

This meta-analysis, including six RCTs with 3521 patients, showed that there was an increase in weight with INSTIs in comparison with other families of antiretrovirals, including follow-up periods from 48 to 96 weeks. Moreover, we found small absolute reductions in all lipid levels (total cholesterol, LDL cholesterol, HDL cholesterol, and triglycerides). The certainty of the evidence was moderate for weight and low for all lipid outcomes.

### 4.2. What Is Known in the Literature about Our Research Question

The mechanisms associated with weight gain in patients using INSTIs may be multiple. Previous research described the inhibition of melacortin-4 receptors (MC4R) by DTG as being associated with weight changes [30]. Another proposed theory is the rapid decrease in HIV RNA seen with INSTIs, given the correlation between HIV RNA and resting energy expenditure, which could be different among different types of ARV [30]. Some associated factors with weight gain could be the degree of virological suppression, the impairment of adipogenesis and adipocyte metabolism, the use of medications to treat neuropsychiatric disorders associated with INSTI use, and the changes in the intestinal microbiome [16,30]. Moreover, weight gain during ART could be associated with an increase in cardiovascular risk, type 2 diabetes (with follow-ups to 18 months), and hypertension (with follow-ups to 12 months) [31,32,33].

Olawepo et al. in 2020 [34] published a meta-analysis of 18 studies, including 15 cohorts and 3 RCTs. All the studies showed an increase in body mass index (BMI) with ART (MD 1.58 kg/m^2^, 95% CI 1.36 to 1.81, I^2 ^= 85%, *p *< 0.01). Subgroup analyses showed MDs of 1.54 kg/m^2^ (95% CI 1.21 to 1.87) and 1.63 kg/m^2^ (95% CI 1.34 to 1.91) for studies with follow-ups of ≤1 year and >1 year, respectively; authors reported that the greatest increase in BMI was during the first 6–12 months of treatment. Bai et al. in 2022 [30] described weight changes in patients who received integrase inhibitors with information from 8 studies (5 cohort studies and 3 RCTs) and analyzed their data with a network meta-analysis. The weight increase was significantly higher with dolutegravir in comparison to elvitegravir (MD 1.13 kg, 95% CI 0.18 to 2.07). Moreover, probabilities of this trend obtained by the cumulative ranked area under the curve (SUCRA) were 79.2% for dolutegravir, 77.9% for bictegravir, 33.2% for raltegravir, and 9.7% for elvitegravir, with dolutegravir being the drug associated with a higher weight increase in comparison to other INSTIs [30]. Pantazis et al. [35] presented the results of a large cohort, including PWHs who started ART in or after 2010. In this publication, the subpopulation with normal BMI at the beginning of the study gained 6 kg, 4 kg, and 3 kg after four years of treatment using INSTIs, boosted PIs, and NNRTIs, respectively. On the other hand, the prevalence of obesity increases from 5.7% to 12.2%, 14.2%, and 18.1% with NNRTIs, boosted PIs, and INSTIs regimens. This author found differences between INSTIs because dolutegravir and raltegravir produce higher BMI increases than elvitegravir [35]. In our study, we found an increase of 2.15 kg, slightly more than the report of Olawepo et al. at 12 months of follow-up and lower than the report of Pantazis et al. at four years of follow-up. However, none of these studies included naïve PWH.

It is important to mention that certain guidelines and real-world studies have described that African Americans, women, and more recently Hispanics, which are disproportionally affected by HIV infection, have a higher risk of weight gain with INSTI-based regimens. Among female African American PWH who were naïve to antiretroviral therapy, the weight gain in 9.5 months of follow-up was 1.5 kg. with INSTIs [36], and in non-naïve PWH, the weight increase was 1.7 kg in females and 0.9 kg among African Americans [37]. However, in this population, switching from members of the INSTI family to members of the protease inhibitor family may reverse the weight gain > 6 months post-switch [37]. With reference to lipid variations, we found a decrease in total cholesterol, LDL cholesterol, and triglycerides with the use of INSTIs in comparison to other families of antiretrovirals. The first published studies with INSTIs, mainly focusing on raltegravir, noted a neutral effect of this drug on lipids [4]. In the SPRING-2 study that compares two INSTIs, raltegravir, and dolutegravir, no changes in fasting lipids were found. [38]. Subsequent RCTs also showed dolutegravir to have a lower effect on lipids in comparison to efavirenz, raltegravir, and the combination of ritonavir with a protease inhibitor, but most of these studies were conducted in non-naïve PWH [39,40,41,42]. Nevertheless, recent evidence reported that switching from boosted protease inhibitors on NNRTI-based regimens to dolutegravir/abacavir/lamivudine generates significant improvements in lipid profiles, including total cholesterol/HDL ratio, and a neutral effect on glycemia at 48 weeks [43].

More information was analyzed in 17 international cohorts with 29432 PWH across Europe and Australia. In this report, participants taking INSTIs had a lower incidence of dyslipidemia compared with those on boosted protease inhibitors (adjusted incidence rate ratio [IRR] 0.71, 95% CI 0.59 to 0.85), but a higher rate compared with those on NNRTIs (IRR 1.35, 95% CI 1.15 to 1.58). Compared with dolutegravir, the incidence of dyslipidemia was higher with elvitegravir/cobicistat (IRR 1.20, 95% CI 1.00 to 1.43) and raltegravir (IRR 1.24, 95% CI 1.02 to 1.51), but lower with rilpivirine (IRR 0.77, 95% CI 0.63 to 0.94) [44]. In a study that included treatment-naïve (n = 179) and treatment-experienced (n = 290) PWH followed by 24 months, the mean changes in total cholesterol (31.6 mg/dL, SD = 40.2, *p *< 0.001) and LDL-cholesterol (18.1 mg/dL, SD = 26.0, *p *< 0.001) were higher for tenofovir alafenamide/emticritabine/elvitegravir/cobicistat in comparison to tenofovir disoproxil fumarate/emtricitabine/elvitegravir/cobicistat and abacavir/lamivudine/dolutegravir. On the other hand, LDL-cholesterol decreased over time in the dolutegravir group, defining differences in lipid variations between this combination of molecules and probably between different class members [45].

In naïve PWH, raltegravir, dolutegravir, and bictegravir were associated with a similar, slight increase in lipids and a superior profile compared with boosted-PI, efavirenz, and elvitegravir [46]. Then, our data confirm the safety profile of the INSTI family on lipid variations, reporting lower values of lipid parameters with its use in a naïve group of PWH.

### 4.3. What Our Study Adds to the Literature

Our study only focused on RCTs in naïve PWH, and we found a weight increase in those who received ART based on INSTIs. We also found small absolute reductions in lipid concentrations in comparison to NNRTI- or PI-based ART. Meta-analyses are scarce in naïve PWH, so this information could be useful to determine the association between INSTI use and increased weight. We only focused on RCTs in our study, whereas previous systematic reviews [30,32] combined cohorts and RCTs. Furthermore, our searches are the most recent of those systematic reviews. Finally, we formally evaluated the risk of bias and certainty of evidence per outcome with the RoB2 tool and GRADE methodology, respectively.

### 4.4. Limitations

Our study has some limitations. First, the number of RCTs that compared the effect of INSTI use with other families of antiretrovirals is scarce. Second, there may be differences between members of the INSTI family, but the small number of RCTs did not allow for comparing individual drugs. Moreover, the most recent studies have compared individual members of the INSTI drug family. Third, there needed to be more information about lipids, weight, and other metabolic parameters in the pivotal studies that are only focused on the efficacy of the drugs on virological control. Fourth, our study only included RCTs with periods of follow-up between 48 and 96 weeks, and we cannot draw any conclusions about the earlier or later effects of the drugs. Fifth, there may be differences in effects among different doses of controls, for example, with different efavirenz formulations (400 mg vs. 600 mg). Sixth, there could be mixed effects on weight gain using combination therapy, as INSTIs are not the unique drugs used for the treatment of PWH. Seventh, as we are analyzing a few studies, weight changes in subpopulations of PWH at higher risk of gaining weight were not described. Finally, the CoE for all lipid outcomes was low and moderate for weight changes.

## 5. Conclusions

In naïve PWH, the use of integrase inhibitor-based therapy in comparison with PI- or NNRTI-based ART was associated with a small increase in weight and small absolute decreases of total cholesterol, LDL cholesterol, HDL cholesterol, and triglycerides. The CoE was moderate for weight and low for all lipid outcomes; there were two RCTs at high risk of bias.

## Figures and Tables

**Table 1:** Characteristics of included randomized controlled trials.

Author	Year	Country	Drugs	Dose Studied	Male Gender	Age	Follow Up
Lennox [24]	2009	Multicentric (Australia, Brazil, Canada, Chile, Colombia, France, Germany, India, Italy, Mexico, Peru, Spain, USA, Thailand)	Raltegravir (n = 281) Efavirenz (n = 282)	400 mg 600 mg	81% 82%	37.6 (9.0) 36.9 (10)	48 weeks
Rockstroh [25]	2013	Multicentric (USA, Canada, Puerto Rico, Australia, Thailand, Austria, Belgium, Denmark, France, Germany, Italy, Netherlands, Portugal, Sweden, Switzerland, UK, Mexico)	Elvitegravir (n = 353) Atazanavir-ritonavir (n = 355)	150 mg 300/100 mg	91.8% 89%	38 (10.5) 39 (9.8)	96 weeks
Calmy [26]	2020	Cameroon	Dolutegravir (n = 310) Efavirenz (n = 303)	50 mg 400 mg	36.5% 31.7%	38 (31–46) 36 (29–43)	96 weeks
Verter [27]	2020	South Africa	Tenofovir alafenamide/emtricitabine + dolutegravir (n = 351) Tenofovir disopropil/emtricitabine/dolutegravir (n = 351) Tenofovir disopropil/emtricitabine/efavirenz (n = 351)	300/200 + 50 mg 300/200/50 mg 300/200/600 mg	41% 39% 43%	32 (8.1%) 33 (7.8%) 32 (7.4%)	96 weeks
Walmsley [28]	2013	Multicentric (Spain, Belgium, Romania, Germany, France, Italy, USA)	Dolutegravir (n = 414) Efavirenz (n = 419)	50 mg 600 mg	84% 85%	36 (18–63) 35 (18–85)	48 weeks
Clotet [29]	2014	Multicentric (Spain, USA, Germany, France, Italy, Romania, Russia, Switzerland)	Dolutegravir (n = 242) Darunavir-ritonavir (n = 242)	50 mg 800/100 mg	87% 83%	35.7 (10.6) 36.2 (10.64)	48 weeks

## Supplementary Materials

The following supporting information can be downloaded at: https://www.mdpi.com/article/10.3390/jcm12113644/s1, Table S1: Search Strategies; Table S2: Summary of Findings: Effects on Study Outcomes.
