# Supplementary material for: Weight and Metabolic Outcomes in Naïve HIV Patients Treated with Integrase Inhibitor-Based Antiretroviral Therapy: A Systematic Review and Meta-Analysis
Source: J Clin Med. 2023 May 24;12(11):3644. doi: 10.3390/jcm12113644 (PMC10253862; doi:10.3390/jcm12113644)
Supplement: Supplementary file 1 [file jcm-12-03644-s001.zip › jcm-2223637-supplementary.pdf]

Journal of
Clinical Medicine

Table S1. Search strategies.

Database	Search terms
PubMed	(HIV[mesh] OR HIV[tiab] OR "HIV-1"[tiab] OR HIV1[tiab] OR "HIV-2"[tiab] OR HIV2[tiab] OR "Acquired Immunodeficiency Syndrome"[mesh] AIDS[tiab] OR "HIV/AIDS"[tiab] OR "human immunodeficiency virus"[tiab] OR "human immunedeficiency virus"[tiab] OR "human immuno-deficiency virus"[tiab] OR "human immune-deficiency virus"[tiab] OR "Acquired Immunodeficiency Syndrome"[tiab] OR "Acquired Immunedeficiency Syndrome"[tiab] OR "Acquired Immuno-deficiency Syndrome"[tiab] OR "Acquired Immune-deficiency Syndrome"[tiab]) AND ("integrase inhibitors"[mesh] OR "integrase inhibitor"[tiab] OR "integrase inhibitors"[tiab] OR Raltegravir[tiab] OR Isentress[tiab] OR Dolutegravir[tiab] OR Tivicay[tiab] OR Bictegravir[tiab] OR Cabotegravir[tiab] OR Elvitegravir[tiab]) AND ("body weight"[mesh] OR weight[tiab] OR "body mass index"[mesh] OR "Body mass index"[tiab] OR BMI[tiab] OR glucose[mesh] OR glucose[tiab] OR "blood pressure"[mesh] OR "blood pressure"[tiab] OR cholesterol[mesh] OR cholesterol[tiab] OR "cholesterol, LDL"[mesh] OR LDL[tiab] OR "cholesterol, HDL"[mesh] OR HDL[tiab] OR triglycerides[mesh] OR triglycerides[tiab]) AND (random*[tiab] OR trial[tiab] OR placebo[tiab])
Embase	(HIV:ti,ab OR "HIV-1":ti,ab OR HIV1:ti,ab OR "HIV-2":ti,ab OR HIV2:ti,ab OR AIDS:ti,ab OR "HIV/AIDS":ti,ab OR "human immunodeficiency virus":ti,ab OR "human immunedeficiency virus":ti,ab OR "human immuno-deficiency virus":ti,ab OR "human immune-deficiency virus":ti,ab OR "Acquired Immunodeficiency Syndrome":ti,ab OR "Acquired Immunedeficiency Syndrome":ti,ab OR "Acquired Immuno-deficiency Syndrome":ti,ab OR "Acquired Immune-deficiency Syndrome":ti,ab) AND ("integrase inhibitor":ti,ab OR "integrase inhibitors":ti,ab OR Raltegravir:ti,ab OR Isentress:ti,ab OR Dolutegravir:ti,ab OR Tivicay:ti,ab OR Bictegravir:ti,ab OR Cabotegravir:ti,ab OR Elvitegravir:ti,ab) AND (weight:ti,ab OR "Body mass index":ti,ab OR BMI:ti,ab OR glucose:ti,ab OR "blood pressure":ti,ab OR cholesterol:ti,ab OR LDL:ti,ab OR HDL:ti,ab OR triglycerides:ti,ab) AND (random*:ti,ab OR trial:ti,ab OR placebo:ti,ab)
Scopus	TITLE-ABS ((HIV OR "HIV-1" OR HIV1 OR "HIV-2" OR HIV2 OR AIDS OR "HIV/AIDS" OR "human immunodeficiency virus" OR "human immunedeficiency virus" OR "human immuno-deficiency virus" OR "human immune-deficiency virus" OR "Acquired Immunodeficiency Syndrome" OR "Acquired Immunedeficiency Syndrome" OR "Acquired Immuno-deficiency Syndrome" OR "Acquired Immune-deficiency Syndrome") AND ("integrase inhibitor" OR "integrase inhibitors" OR Raltegravir OR Isentress OR Dolutegravir OR Tivicay OR Bictegravir OR Cabotegravir OR Elvitegravir) AND (weight OR "Body mass index" OR BMI OR glucose OR "blood pressure" OR cholesterol OR LDL OR HDL OR triglycerides) AND (random* OR trial OR placebo))

Table S2. Summary of findings table of effects of integrase inhibitors on study outcomes.

			Anticipated absolute effects	
			**Risk with NNRTI / protease inhibitors**	**Risk difference with Integrase inhibitors**
Weight<br>assessed with: Weight in kilograms<br>follow-up: mean 96 weeks	1315<br>(2 RCTs)	⨁⨁⨁◯<br>Moderate[a]	The mean weight was **2.81** kg	MD **2.15 kg higher**<br>(1.4 higher to 2.9 higher)
Total cholesterol (TC)<br>follow-up: mean 96 weeks	2819<br>(5 RCTs)	⨁⨁◯◯<br>Low[b,c]	The mean total cholesterol was **25.76** mg/dL	MD **13.44 mg/dL lower**<br>(23.49 lower to 3.39 lower)
LDL cholesterol (LDL-c)<br>assessed with: mean<br>follow-up: mean 96 months	1499<br>(3 RCTs)	⨁⨁◯◯<br>Low[d,e]	The mean LDL cholesterol was **20.54** mg/dL	MD **11.37 mg/dL lower**<br>(19.24 lower to 3.5 lower)
HDL cholesterol (HDL-c)<br>assessed with: mean<br>follow-up: mean 96 months	1499<br>(3 RCTs)	⨁⨁◯◯<br>Low[d,f]	The mean HDL cholesterol was **10.62** mg/dL	MD **5.03 mg/dL lower**<br>(10.61 lower to 0.54 higher)
Triglycerides (TG)<br>assessed with: mean<br>follow-up: mean 96 weeks	2819<br>(5 RCTs)	⨁⨁◯◯<br>Low[b,g]	The mean triglycerides was **49.40** mg/dL	MD **20.7 mg/dL lower**<br>(37.25 lower to 4.15 lower)

Explanations

a. Verter had high ROB in the domain of randomisation process and moderate ROB in the domain of deviations from the intended interventions. Calmy had moderate ROB in the domains of randomisation process, deviations from the intended intervention and selection of the reported result.

b. Clotet had high ROB in the domains of deviations from the intended interventions and selection of the reported result.. Calmy had moderate ROB in the domains of randomisation process, deviations from the intender intervention and selection of the reported result. Rockstroh had moderate ROB in the domain of randomisation process

c. High heterogeneity: I^2=96%

d. Clotet had high ROB in the domains of deviations from the intended interventions and selection of the reported result

e. High heterogeneity: I^2=83%

f. High heterogeneity: I^2=95%

g. High heterogeneity: I^2=92%
